# Determinants of primary healthcare services utilisation in an under-resourced rural community in Enugu State, Nigeria: a cross-sectional study

**DOI:** 10.11604/pamj.2022.42.209.33317

**Published:** 2022-07-15

**Authors:** Ugochukwu Uzoechina Nwokoro, Onyekachi Michael Ugwa, Adanma Chidinma Ekenna, Izuchukwu Frank Obi, Chinemerem Daniel Onwuliri, Chuka Agunwa

**Affiliations:** 1 Department of Community Medicine, University of Nigeria Teaching Hospital, Enugu, Nigeria,; 2 Nigeria Field Epidemiology and Laboratory Training Program, Abuja, Nigeria,; 3 Department of Community Medicine, College of Medicine, University of Nigeria Enugu Campus, Enugu, Nigeria

**Keywords:** Primary healthcare, health services utilisation, Enugu State, Southeast Nigeria

## Abstract

**Introduction:**

primary health care (PHC) is essential towards achieving universal health coverage. Improving PHC services require understanding context-specific factors influencing utilisation. We assessed the factors influencing utilisation of PHC services in a rural community in Enugu, Nigeria.

**Methods:**

we conducted a cross-sectional community-based survey between May and June, 2017. Information on socio-demographic characteristics, utilisation of PHC services, community- and PHC facility-related factors associated with utilisation of PHC services was obtained from 335 adult residents aged ≥ 18 years using a pre-tested semi-structured interviewer-administered questionnaire. Data were analysed using descriptive and inferential statistics at 5% level of significance.

**Results:**

of the 335 respondents, 155 (46.2%) reported utilisation of PHC services the last time they were sick. Of 178 respondents who did not utilise PHC services, 51 (28.7%) reported poor quality health services, 41 (23.0%) unavailability of medical doctors, 31 (17.4%) long patient waiting time and 25 (14.0%) unavailability of drugs as reasons for non-utilisation. Being a female (AOR = 2.3 (95% CI 1.3 - 4.0)), affordability of health services (AOR = 2.4 (95% CI 1.3 - 4.6)), inadequacy of healthcare staff (AOR = 0.3 (95% CI 0.1 - 0.5)), shorter hospital waiting time (AOR = 2.2 (95% CI 1.2 - 4.3)) and satisfaction with PHC services during previous visit (AOR = 2.6 (95% CI 1.1 - 6.3)) influenced utilisation of PHC services.

**Conclusion:**

PHC services utilisation was low. Improving utilisation would require addressing cost of health services, adequacy of healthcare staff, patient waiting time and ensuring patient satisfaction with PHC services.

## Introduction

Primary health care (PHC) was first recognized globally in 1978 as a veritable tool for achieving health for all peoples of the world and for addressing the main health problems in the community by providing promotive, preventive, curative and rehabilitative services to the populace [1]. It represents the first contact individuals, families and the community make with the national health system in the health care spectrum [1]. In 2008, the World Health Organization (WHO) not only re-emphasized the importance of PHC as the benchmark for providing comprehensive health services globally and nationally in a safe, effective and socially productive manner but also echoed the need to adopt a people oriented approach [2].

Following the renewed commitment to health and well-being for all by the United Nations (UN) in 2015, PHC further gained traction as an effective, efficient and equitable approach towards enhancing health, thus making it an important tool towards achieving Universal Health Coverage (UHC) [3]. The Sustainable Development Goal (SDG) 3 addresses all major health priorities, aiming to ensure healthy lives and promote well-being for all by the year 2030 [4]. Primary health care continues to be recognized globally as an important approach towards achieving the health-related SDGs and UHC [5,6]. As the discourse on achieving UHC continues to gain momentum, the significant contribution of PHC to health and health systems in Low and Middle Income Countries (LMIC) has been highlighted [7].

In Nigeria, successive national health policies recognize PHC as a core underlying principle, serving as the basic philosophy and strategy for national health development [8,9]. Since the adoption of PHC as the cornerstone of the health system in Nigeria, it has undergone series of evolution with various steps taken to reposition and revitalize it towards improving access to and utilisation of basic health services [10]. The implementation of PHC in Nigeria is through services offered at various primary health care facilities and health posts across the country. These services include health education concerning prevailing health problems and their prevention and control, maternal and child health services including family planning, immunization against the major infectious diseases as well as treatment of minor ailments. Despite repeated attempts by the Nigerian government to reposition and revitalize PHC, various constraints have hampered its implementation and thus threaten the achievement of UHC, with poor or low utilisation of PHC services being a major factor [11]. The preference of unorthodox treatment sites such as patent medicine vendors and traditional healers, as well as private and government-owned secondary and tertiary health facilities other than the designated primary health care facilities by majority of populations in parts of Nigeria has been documented [12,13].

In order for countries to ensure the delivery and utilisation of high quality and safe PHC services, the in-country choice of specific interventions should not only be informed by global but also local evidence driven by context-specific PHC-oriented research [14]. Understanding the factors influencing utilisation of PHC services especially in developing regions would help guide policy formulation towards improving uptake of health services at the primary health care level, thus contributing towards achieving UHC and ensuring health and well-being, particularly among vulnerable populations and rural communities in poor-resource settings [15,16]. This study therefore aimed to determine PHC services utilisation and to assess community as well as PHC facility-related factors associated with utilisation of PHC services among adult residents of a rural community in Nsukka local government area of Enugu State, Nigeria.

## Methods

**Study area:** the study was conducted in Obukpa, a rural community in Nsukka local government area of Enugu State, South-East Nigeria. Obukpa comprises four autonomous communities namely: Owerre, Obige, Ogbagu and Ajuona with a total of 57 villages. The study area is inhabited mostly by locals whose major occupations are farming and petty trading. Primary health care services are provided to the community residents by a comprehensive health center located at Obige-Obukpa and five health posts located across the four autonomous communities. The comprehensive health center is a 30 bed facility with a total of 75 medical and non-medical staff including a medical doctor. Primary health care services offered at the facility include health education, maternal and child health services including family planning, treatment of locally endemic and epidemic diseases, primary dental care, basic laboratory and pharmaceutical services.

**Study design:** the study was a cross-sectional community-based household survey conducted between May and June, 2017.

**Study population:** the study population comprised adult residents aged 18 years and above in Obukpa community, Nsukka local government area of Enugu State.

**Sample size determination and sampling procedure:** a minimum sample of 260 community residents was calculated using the Leslie Kish formula based on the assumption that utilisation of PHC services by a rural population in Southeastern Nigeria is 18.9% [13], design effect of 2, precision of 5%, and adjusting for a non-response rate of 10%. A two-stage cluster sampling technique was used to recruit study participants. First stage involved selection of one village from each of the four autonomous communities in Obukpa using simple random sampling, giving a total of four selected villages namely Ogwashi-Ujom, Obigeti-Akpogu, Aguohuru, and Amaeze-Uwani. In each selected village, all adults aged 18 years and above from all households, who gave informed consent were included in the study.

**Study instrument and data collection method:** a pre-tested semi-structured interviewer administered questionnaire adapted from the World Bank´s working paper on improving primary health care delivery in Nigeria and partly developed from review of other literature [12,17] and consisting of the following sections: socio-demographic characteristics, utilisation of PHC services the last time sick, reasons for not utilizing PHC services, community- and PHC facility-related factors associated with utilisation of PHC services, was used to obtain information from the selected respondents. The questionnaire was prepared in English and translated to the local language, Igbo. The data collectors (four trainee community health officers and two resident doctors in the department of community medicine) speak the local language fluently and were trained for two days on the study protocol, questionnaire, interview techniques, approach to household heads and adult household members, double-checking of filled questionnaires and data entry into an electronic database.

**Data analysis:** data was coded and statistical analysis conducted using Microsoft Excel and Epi info version 7.1.5.2 software. Frequencies and percentages were computed as descriptive statistics. At bivariate analysis, association between utilisation of PHC services and independent variables such as age, sex, educational status, occupational status, presence of a child younger than five years in the household, round trip time to reach primary health care facility, affordability of PHC services, perceived adequacy of healthcare staff, perceived adequacy of available medical equipment and ancillary services, hospital waiting time and satisfaction with health services were determined using Chi-square tests with odds ratios and corresponding 95% confidence intervals computed. Variables that had a p-value of ≤ 0.2 at bivariate analysis were entered into the logistic regression model to determine the predictors of PHC services utilisation. The results of the logistic regression were reported using adjusted odds ratios and 95% confidence intervals. All statistical analyses were performed at 5% level of significance.

**Ethical considerations:** ethical approval for this study was obtained from the Health Research Ethics Committee of the University of Nigeria Teaching Hospital, Ituku-Ozalla Enugu State (reference: UNTH/CSA/329/OL.5). Permission to conduct the study was sought and obtained from the traditional leaders of the autonomous communities. Written informed consents were obtained from all study participants after they have demonstrated understanding of study procedures, risks and voluntariness. Confidentiality of all the participants was assured and maintained during and after the study.

## Results

**Socio-demographic characteristics of respondents:** there were a total of 335 adult respondents from 103 households in the survey. The median age (IQR) of respondents was 38.0 (25.0 - 55.0) years. One hundred and twenty (35.8%) respondents were younger than 30 years while 66 (19.7%) were aged 60 years and above. Majority, 212 (63.3%) of the respondents were females, 175 (52.2%) were married and 82 (24.5%) have no formal education (Table 1).

**Table 1:** socio-demographic characteristics of study participants, Enugu State, Nigeria

Variable (N = 335)	Frequency	Percent
**Age (years)**		
18 - 29	120	35.8
30 - 39	52	15.6
40 - 49	48	14.3
50 - 59	49	14.6
≥ 60	66	19.7
**Sex**		
Female	212	63.3
Male	123	36.7
**Marital status**		
Single	114	34.0
Married	175	52.3
Divorced	2	0.6
Widowed	44	13.1
**Educational level**		
No formal education	82	24.5
Primary	51	15.2
Secondary	153	45.7
Tertiary	49	14.6
**Occupational status**		
Employed	252	75.2
Unemployed	83	24.8

**Utilisation of primary health care services:** of the 335 respondents, 155 (46.2%) utilised health services at the primary health care facilities in the community the last time they were sick.

**Reasons for not utilizing primary health care services at designated PHC facilities:** one hundred and seventy-eight respondents gave reasons for not utilizing PHC services which include: perceived poor quality of health services (28.7%), unavailability of medical doctors (23.0%), long patient waiting time (17.4%) and unavailability of drugs (14.0%) (Table 2).

**Table 2:** respondents' reasons for not using primary healthcare services, Enugu State, Nigeria

Reason	Frequency	Percent
Poor quality service	51	28.7
Unavailability of doctors	41	23.0
Long patient waiting time	31	17.4
Unavailability of drugs	25	14.0
Health services unaffordable	8	4.5
Others	22	12.4
Total	178	100.0

**Factors influencing utilisation of primary health care services:** at bivariate analysis, patient-related factors such as sex, respondents having a child younger than five years in their household and satisfaction with health services during previous visit as well as health facility-related factors like cost of health services, round trip time to the PHC facility, perceived adequacy of healthcare staff, hospital waiting time, availability of medical equipment and ancillary services were associated with utilisation of PHC services. However, at multivariate analysis, being a female (AOR = 2.3 (95% CI 1.3 - 4.0)), cost of health services perceived as affordable (AOR = 2.4 (95% CI 1.3 - 4.6)), health care staff perceived to be inadequate (AOR = 0.3 (95% CI 0.1 - 0.5)), hospital waiting time of shorter than one hour (AOR = 2.2 (95% CI 1.2 - 4.3)) and satisfaction with health services during previous visit (AOR = 2.6 (95% CI 1.1 - 6.3)) predicted utilisation of PHC services (Table 3).

**Table 3:** factors influencing primary healthcare services utilisation in an under-resourced rural community in Enugu State, Nigeria

Variables	Utilisation of PHC services		Crude OR (95% CI)	P-value	Adjusted OR (95% CI)
	Yes n (%)	No n (%)		(Bivariate)	
**Age (years)**					
< 60	128 (47.6)	141 (52.4)	1.3 (0.8 - 2.3)	0.33	NA
≥ 60	27 (40.9)	39 (59.1)	1		
**Sex**					
Female	109 (51.4)	103 (48.6)	1.8 (1.1 - 2.8)	0.01	2.3 (1.3 - 4.0)
Male	46 (37.4)	77 (62.6)	1		
**Educational status**					
Formal education	116 (45.9)	137 (54.1)	0.9 (0.6 - 1.5)	0.79	NA
No formal education	39 (47.6)	43 (52.4)	1		
**Occupational status**					
Employed	121 (48.0)	35 (41.7)	1.3 (0.8 - 2.2)	0.26	NA
Unemployed	34 (41.9)	143 (58.1)	1		
**Child <5 years in Household**					
Yes	49 (58.3)	35 (41.7)	1.9 (1.2 - 3.2)	< 0.01	1.2 (0.7 - 2.2)
No	103 (41.9)	143 (58.1)	1		
Round trip time to HF*					
< 1 hour	97 (41.8)	135 (58.2)	0.6 (0.3 - 0.9)	0.01	0.6 (0.3 - 1.1)
≥ 1 hour	58 (56.3)	45 (43.7)	1		
**Perceived cost of HS†**					
Affordable	127 (60.5)	83 (39.5)	5.3 (3.2 - 8.8)	< 0.001	2.4 (1.3 - 4.6)
Not affordable	28 (22.4)	97 (77.6)	1		
**Health care staff**					
Not adequate	30 (20.7)	115 (79.3)	0.1 (0.08 - 0.23)	<0.001	0.3 (0.1 - 0.5)
Adequate	125 (66.5)	63 (33.5)	1		
**Medical equipment and ancillary services**					
Adequate	126 (62.1)	77 (37.9)	5.8 (3.5 - 9.6)	< 0.001	1.4 (0.7 - 2.9)
Not adequate	29 (22.0)	103 (78.0)	1		
**Hospital waiting time**					
< 1 hour	125 (51.9)	116 (48.1)	2.3 (1.4 - 3.8)	< 0.001	2.2 (1.2 - 4.3)
≥ 1 hour	30 (31.9)	64 (68.1)	1		
**Satisfaction with HS†**					
Yes	146 (56.2)	114 (43.9)	9.4 (4.5 - 19.7)	< 0.001	2.6 (1.1 - 6.3)
No	9 (12.0)	66 (88.0)	1		

* HF: healthcare facility; †HS: healthcare services; NA: not applicable

## Discussion

This study found primary health care services utilisation among adults to be low at 46.2%. The PHC services utilisation found in this study is however much higher than the 7.5% and 18.9% utilisation reported by studies in Northwest and Southeast Nigeria respectively [12,13] but lower than the 76.8%, 89.4% and 89.5% utilisation reported by studies in South-South, North-Central and Southwest Nigeria respectively [18-20]. The low utilisation of primary health care services observed in this study may be an indicator of the low confidence that the people have in the services offered at the primary health care level in the study setting, thus making them seek primary care at higher level hospitals or other places. Studies in Nigeria have reported majority of households preferring to utilise patent medicine vendors or pharmacies other than primary health care facilities as the first line of therapy [12,13]. The preference for patent medicine stores as the first choice when seeking health care services has been suggested to be an indicator that the people utilise health services for curative as opposed to preventive purposes [12].

The main reasons reported by respondents for not utilizing PHC services in this study were perceived poor quality health services, unavailability of doctors and drugs, long patient waiting time and high cost of services at the primary health care facilities. Previous studies in Nigeria have reported these as reasons for non-utilisation of primary health care services in their settings [12,13]. Equally another study in Southeast Nigeria reported long waiting queues and lack of doctors, amongst other reasons, as major factors discouraging utilisation of maternal and child health services at the primary health care level [21]. Patient waiting time and availability of essential drugs have also been reported to be important indicators of patient satisfaction with primary health care services [22].

Socio-demographic factors such as age category, educational level, occupational status and having a child younger than five years in a respondent´s household were not significantly associated with utilisation of primary health care services. However, respondents´ sex was found to exert significant influence on utilisation of primary health care services. Women had 80% higher odds of utilizing primary health care services in this study. The reason for this finding is unclear as socio-cultural practices and beliefs in many developing regions including Africa often make women unable to make decisions including those of seeking appropriate health care independent of their male spouses. However, it might be because the services offered at the primary health care facilities in the study setting such as maternal and child health services including family planning and immunization services are more likely to attract women to the health facilities compared to men. Previous studies have reported women to have higher health service utilisation compared to men due to gender differences in morbidity patterns and because women are more likely to report their health problems or use preventive health services than men [23-25].

Time taken to reach location of health facility and perceived adequacy of available medical equipment, as well as ancillary services at primary health care facilities were not significantly associated with utilisation of primary health care services in this study. Time taken to reach a health facility relates to the distance separating health services users and the nearest primary health care facility. This serves as a proxy measure of accessibility of primary health care services to community members. Distance has been reported as barrier to utilisation of PHC services especially in rural areas in Nigeria and elsewhere [26,27]. Furthermore, the additional need to take care of transportation and its cost by residents of rural areas has been noted to constitute a barrier to accessing health services [28]. Although community residents who perceived available medical equipment and ancillary services at primary health care facilities to be adequate were more likely to utilise PHC services compared to those who perceived them to be inadequate the influence of this consideration was not significant.

One of the cardinal aims of primary health care is to ensure access to quality and affordable health services needed by individuals, families and the community without catastrophic financial expenditure. This study found that community members who reported cost of health services at the PHC facilities to be affordable were twice as likely to utilise PHC services compared to those reporting health service costs as being unaffordable. High cost of health services has been reported to be a disincentive for utilisation of PHC services [12,19] with individuals in low socioeconomic status groups being the least likely to utilise PHC services [29].

This study also identified community perception of adequacy of medical staff both in terms of staff strength and professional qualification to be a significant determinant of PHC services utilisation. Community members who perceived healthcare staff to be inadequate were less likely to utilise PHC services compared to those who considered the manpower to be adequate in the facilities. Other studies in Nigeria have documented inadequacy of medical staff including lack of doctors as barriers to utilisation of PHC services [12,21]. Doctors are generally perceived by rural dwellers as having the greatest capacity for health care delivery. This understanding may influence seeking of care at private and higher tier health facilities by community members without realizing that other primary health care workers are able to address most health challenges with the option of referral where necessary. Hospital waiting time was also found to be a significant factor influencing utilisation of PHC services. Community residents who reported experiencing shorter waiting time were more likely to utilise PHC services. Previous studies have reported long waiting times to be a predictor of PHC services utilisation and satisfaction with health services [19,21,22,30].

In this study, satisfaction with previous PHC services was observed to be a predictor of utilisation of PHC services. This is similar to findings from previous studies [20,21] with patients who were not satisfied with PHC services reporting an unwillingness to return to PHC clinics for subsequent visits [31]. Patient satisfaction is a measure of perceived quality of health service received. Gaps in health services user experience and quality still exist in primary care especially in Low and Middle Income Countries (LMICs) [32]. Addressing these gaps would contribute in improving PHC services utilisation.

**Limitations of study:** this study has provided insight into some important context-specific factors influencing PHC services utilization in a resource-limited setting. However, the study has a number of limitations. This study being a cross sectional survey that relied on respondents´ memory of experiences at the health facility the last time they utilized health services there may have been prone to recall bias. Furthermore, community residents´ report of satisfaction with services received may have been prone to social desirability bias whereby respondents report satisfaction with health services received to favour the health facility utilized.

## Conclusion

Primary health care services utilisation was low in the study setting. Community level factors such as respondents´ sex and satisfaction with primary health care services and health facility-related factors such as cost of health services, adequacy of healthcare staff and long patient waiting time were important determinants of PHC services utilisation. Improving utilisation would require addressing cost of health services, adequacy of healthcare staff, patient waiting time and ensuring patient overall satisfaction with PHC services.

### What is known about this topic

- *Primary Healthcare (PHC) is an important approach toward the achievement of universal health coverage as it brings healthcare to the doorsteps of the people;*
- *PHC services utilisation varies in different settings and factors influencing utilisation vary in different settings*.

### What this study adds

- *Utilisation of PHC services was low in Obukpa community Southeast Nigeria;*
- *predictors of PHC service utilisation include: client being female, client satisfaction with services, short clinic waiting time and affordable services*.
